# Trends in the Link Between Perceived Social Support and Life Satisfaction in Adolescents (2013/14–2021/22): A Cross-National Study

**DOI:** 10.3389/ijph.2024.1607283

**Published:** 2024-07-10

**Authors:** Romain Brisson, Jana Furstova, Lenka Sokolová, Charli Eriksson, Meyran Boniel-Nissim, Petr Badura

**Affiliations:** ^1^ Centre for Childhood and Youth Research, University of Luxembourg, Esch-sur-Alzette, Luxembourg; ^2^ Olomouc University Social Health Institute (OUSHI), Palacký University Olomouc, Olomouc, Czechia; ^3^ Institute of Applied Psychology, Faculty of Social and Economic Sciences, Comenius University, Bratislava, Slovakia; ^4^ National Centre for Suicide Research and Prevention, Department of Learning, Informatics, Management and Ethics (LIME), Karolinska Institutet (KI), Solna, Sweden; ^5^ The Max Stern Academic College of Emek Yezreel, Israel

**Keywords:** perceived social support, trends, life satisfaction, adolescents, HBSC

## Abstract

**Objectives:**

This repeated cross-sectional study aimed to (a) report trends in adolescents’ perceived family, friend, classmate, and teacher support, (b) estimate the extent to which each source of support related to life satisfaction across space and time, and (c) ascertain whether sociodemographic factors moderated the relationship in question.

**Methods:**

We relied on data pertaining to the 2013/14, 2017/18, and 2021/22 waves of the *Health Behaviour in School-aged Children* study. The examined sample covered 44 countries and regions (*n* = 716,083; *M*_AGE_ = 13.6; *SD*_AGE_ = 1.64; 50.7% female).

**Results:**

The level of all sources of perceived social support slightly decreased over the examined period (all ω^2^ < .01). Family support involved the largest association with life satisfaction (β = 0.16); friend support, the lowest one (β = 0.03). These associations varied only tenuously across space and time. Sociodemographic factors moderated the link between perceived social support and life satisfaction to a negligible-to-weak extent.

**Conclusion:**

Levels of perceived social support and their associations with life satisfaction subtly changed. Future research may attempt to pinpoint the macrosocial levers of these temporal dynamics.

## Introduction

Research on the correlates and determinants of adolescents’ wellbeing and health has long identified perceived social support (PSS) as a salutogenic factor [1–3]. Although the construct of social support has been defined and operationalized in multiple ways since its extensive use in social science in the 1970s [4, 5], PSS generally designates the self-valuation of the availability of assistance resources among one’s social network [6, 7]. PSS thus differs from received or enacted social support. Moderately correlated with each other [8, 9], PSS and received social support differently relate to wellbeing and health issues [10, 11]. Studies of the associations of received social support with wellbeing and health outcomes have reported contradictory findings [10, 11]. By contrast, PSS has consistently shown positive associations with life satisfaction (LS) [12, 13] and negative associations with adverse outcomes such as anxiety and depression [3, 14], with meta-analyses reporting small-to-moderate effect sizes [10, 15].

While the protective role of PSS against adolescents’ ill-being is well-established, the structural, sociodemographic, and temporal dynamics of this relationship are unclear. Notably, the extent to which all sources of PSS (e.g., family, peers) are linked to wellbeing and health remains open to question [13]. In effect, the widespread examination of one or two sources of PSS [15, 16] and the common conduct of source-per-source (meta-)analyses [17] have limited (a) the assessment of the unique contribution of sources of PSS to wellbeing and health and (b) the identification of potential cumulative or compensatory mechanisms [18, 19]. Furthermore, the studies examining several sources of PSS concomitantly have reported discrepant findings. While PSS from parents or family has generally been found to involve the largest associations with wellbeing and health outcomes [13, 17], findings on other sources have diverged. As an illustration, Stewart and Suldo’s study [20] revealed a link between PSS from classmates and LS but not between PSS from teachers and LS. Heng et al.’s study [21] highlighted the exact reverse pattern.

In a similar vein, research on the moderating role of sociodemographic factors on the link between PSS and wellbeing or health outcomes has engendered contradictory results. For instance, Rueger et al.’s meta-analysis [15] of the link between PSS and depression found no sex differences. The systematic review by Gariépy et al. [22] did find PSS from family to be more protective from depression in girls than in boys, corroborating the findings from Chu et al.’s meta-analysis [10] of the relationship between social support and wellbeing. As noted by Rueger et al. [15], these inconsistencies may reflect methodological and conceptual differences in the meta-analytic and reviewing processes. It should also be noted that family structure and socioeconomic status (SES) have not been systematically considered in this research area. Because of the interdependence of SES and family structure [23], and because family structure has been shown to relate to the family facet of adolescents’ LS [24], the frequent neglect of such potential moderators of the association between PSS and wellbeing is detrimental to the field [25].

Despite decades of research on PSS [4, 5], only a few studies assessed PSS diachronically based on a repeated cross-sectional design [26] or on cross-temporal meta-analysis [27]. To our knowledge, only one study investigated the temporal trends in the link between PSS and LS in adolescents. Scrutinizing the years 2009–2018, Su et al. [28] found the prevalence of adolescents exhibiting a high level of PSS and the association between PSS and LS to fluctuate between 2009 and 2018. The dearth of such studies, however, does not permit to ascertain whether PSS levels and their associations with wellbeing vary over time and with macrosocial changes (e.g., digitalization of social life, divorce normalization, economic recession).

The present study aimed to examine the structural, sociodemographic, and temporal dynamics of the association between PSS and LS, a key indicator of subjective wellbeing [29]. The reliance on data from the *Health Behaviour in School-aged Children* (HBSC) survey allowed us to examine 44 countries and regions across three waves of data collection—2013/14, 2017/18, and 2021/22. Our study had three main research goals. First, to report trends in four sources of PSS, namely, PSS from family, friends, classmates, and teachers. Second, to assess the extent to which distinct sources of PSS related to LS in space and over time. Third, to investigate a potential moderating role of age, family structure, gender, and SES in the relationship between the examined sources of PSS and LS. Given the paucity of research on the temporal link between distinct sources of PSS and adolescents’ LS, the present study should be considered exploratory.

## Methods

### Study Sample

We relied on data pertaining to the 2013/14, 2017/18, and 2021/22 waves of the HBSC study (https://hbsc.org/). Each wave involved a different number of countries and regions—Flanders and Wallonia, two Belgian administrative entities, participated in the study separately. Owing to its focus on temporal dynamics, the present study involved only the countries and regions that partook in at least two waves of data collection (*n* = 44). Consequently, the conducted analyses did not include data from Azerbaijan, Cyprus, Georgia, Kyrgyzstan, Tajikistan, and Turkey. These data are available online (https://data-browser.hbsc.org).

The final sample involved 716,083 adolescents (*M*_AGE_ = 13.6; *SD*_AGE_ = 1.64; age range: 10–16.5; 50.7% female). Sociodemographic characteristics of the sample are reported in Table 1.

**TABLE 1:** Sociodemographic characteristics of the study sample (Health Behaviour in School-aged Children study, 44 countries and regions, 2013/14–2021/22).

	Wave 2013/14					Wave 2017/18					Wave 2021/22				
	*n*	*M*_AGE_	*SD*_AGE_	% ♀	% IF	*n*	*M*_AGE_	*SD*_AGE_	% ♀	% IF	*n*	*M*_AGE_	*SD*_AGE_	% ♀	% IF
Albania	5,024	13.52	1.67	51.0	93.3	1,765	13.55	1.52	54.5	91.5	5,454	13.40	1.73	52.1	87.5
Armenia	3,679	13.13	1.61	52.2	90.3	4,717	13.51	1.64	50.1	89.9	4,346	13.11	1.62	51.3	87.7
Austria	3,458	13.41	1.68	53.4	75.4	4,129	13.28	1.62	50.7	73.4	5,178	13.86	1.64	54.4	70.3
Bulgaria	4,796	13.70	1.68	47.4	76.3	4,548	13.53	1.65	51.6	77.2	3,006	14.01	1.72	49.3	69.6
Canada	12,931	13.82	1.47	50.4	66.3	12,950	13.65	1.47	50.9	69.9	12,586	13.40	1.50	50.2	71.6
Croatia	5,741	13.63	1.64	49.8	84.0	5,169	13.80	1.70	49.0	83.7	5,338	13.56	1.63	51.7	90.6
Czechia	5,082	13.44	1.66	52.4	68.3	11,564	13.37	1.66	49.7	70.9	12,906	13.46	1.65	49.4	68.0
Denmark	3,891	13.71	1.63	53.4	72.2	3,181	13.33	1.61	51.4	73.6	5,255	13.62	1.61	51.2	73.0
England	5,335	13.50	1.67	48.1	70.0	3,397	13.42	1.48	46.6	68.2	4,241	13.73	1.50	51.3	70.4
Estonia	4,057	13.75	1.62	49.7	66.3	4,725	13.78	1.64	49.9	67.9	4,862	13.64	1.63	49.6	67.3
Finland	5,925	13.78	1.67	50.8	70.3	3,146	13.92	1.61	50.3	74.3	3,522	13.64	1.63	50.9	65.9
Flanders	4,393	13.63	1.76	45.2	70.8	4,333	13.38	1.72	50.5	69.5	9,567	13.79	1.64	49.9	75.1
France	5,691	13.47	1.61	49.6	69.9	9,170	13.30	1.46	50.5	68.4	5,280	13.69	1.56	48.4	73.6
Germany	5,961	13.46	1.65	49.1	74.4	4,347	13.41	1.68	53.0	73.4	6,475	13.44	1.67	51.5	73.9
Greece	4,141	13.63	1.63	50.2	84.4	3,863	13.82	1.66	50.1	81.3	6,250	13.51	1.70	52.9	80.9
Greenland	1,020	13.48	1.62	52.2	53.8	1,243	13.17	1.54	52.1	54.9	1,667	13.20	1.63	53.0	51.4
Hungary	3,935	13.40	1.64	50.2	69.9	3,789	13.52	1.63	52.8	71.1	4,068	13.72	1.76	51.9	68.9
Iceland	10,602	13.60	1.63	49.9	69.5	6,996	13.60	1.63	49.8	70.9	9,898	13.56	1.66	48.1	70.9
Ireland	4,098	13.73	1.57	61.1	77.3	3,833	13.41	1.56	49.4	76.8	3,624	13.41	1.59	56.7	78.5
Israel	6,193	13.69	1.64	51.3	84.8	7,712	13.63	1.59	54.8	82.9	8,272	14.23	1.71	52.4	86.5
Italy	4,072	13.66	1.63	49.7	82.5	4,144	13.68	1.62	51.8	79.9	4,572	13.75	1.65	48.2	81.4
Kazakhstan	DNP	DNP	DNP	DNP	DNP	4,868	13.25	1.69	49.6	69.5	7,369	13.43	1.67	52.3	71.9
Latvia	5,557	13.59	1.62	52.3	65.2	4,412	13.47	1.65	50.4	63.3	5,920	13.46	1.65	49.9	63.0
Lithuania	5,730	13.56	1.61	49.2	71.0	3,797	13.70	1.65	49.6	69.0	4,949	13.92	1.68	49.5	68.0
Luxembourg	3,318	13.56	1.62	52.8	71.0	4,070	13.49	1.68	49.9	68.1	4,232	13.62	1.64	49.4	68.9
Macedonia	4,218	13.67	1.60	49.9	88.0	4,658	13.56	1.63	51.1	88.7	4,061	13.74	1.70	52.1	78.8
Malta	2,265	13.54	1.62	48.6	85.9	2,576	13.44	1.63	51.9	77.6	3,407	13.39	1.58	50.2	75.9
Moldova	4,648	13.56	1.66	49.5	79.1	4,686	13.55	1.68	49.9	73.8	5,491	13.68	1.65	49.5	71.0
Netherlands	4,301	13.49	1.59	50.8	76.0	4,698	13.51	1.60	51.3	78.0	4,316	13.37	1.61	48.4	76.8
Norway	3,422	13.35	1.66	51.2	74.6	3,127	13.02	1.61	51.5	71.8	3,260	13.47	1.58	48.6	70.3
Poland	4,545	13.57	1.65	50.2	78.6	5,224	13.59	1.66	50.8	78.4	5,395	13.86	1.68	51.9	78.1
Portugal	4,989	13.49	1.54	52.5	73.8	6,126	13.31	1.53	52.2	71.9	5,182	13.81	1.63	53.2	71.5
Romania	3,980	13.21	1.67	52.8	77.2	4,567	13.20	1.63	51.2	63.2	8,382	13.59	1.61	51.8	72.1
Russia	4,716	13.51	1.56	56.2	68.2	4,281	13.82	1.66	52.3	69.2	DNP	DNP	DNP	DNP	DNP
Scotland	5,932	13.66	1.64	49.9	66.1	5,021	13.52	1.63	51.8	65.9	4,354	13.56	1.58	48.5	69.0
Serbia	DNP	DNP	DNP	DNP	DNP	3,933	13.98	1.68	50.5	77.3	3,713	13.78	1.65	52.7	74.5
Slovakia	6,099	13.48	1.50	49.7	76.4	4,785	13.32	1.52	48.7	75.8	5,584	13.31	1.56	48.4	0.0
Slovenia	4,997	13.62	1.62	51.0	79.6	5,667	13.59	1.63	49.2	81.6	6,327	13.69	1.64	52.3	82.6
Spain	11,136	13.58	1.61	50.8	79.2	4,320	13.62	1.62	51.7	79.9	4,104	13.69	1.61	51.6	74.7
Sweden	7,700	13.60	1.71	50.2	68.6	4,185	13.63	1.64	50.3	70.9	4,379	13.60	1.71	49.8	73.5
Switzerland	6,634	13.50	1.59	50.6	77.1	7,510	13.42	1.60	49.6	78.3	7,141	13.47	1.61	51.5	0.0
Ukraine	4,552	13.66	1.70	52.6	73.7	6,660	13.40	1.63	51.2	73.2	DNP	DNP	DNP	DNP	DNP
Wales	5,154	13.64	1.60	49.0	62.0	15,951	13.52	1.60	49.9	67.9	36,973	13.57	1.64	49.3	66.5
Wallonia	5,892	13.46	1.66	50.3	66.6	5,578	13.28	1.68	50.0	65.9	5,946	13.42	1.68	51.0	67.9
Sample	219,810	13.57	1.63	50.7	74.2	229,421	13.51	1.62	50.7	73.5	266,852	13.60	1.65	50.6	72.8

Notes: IF = “intact families”; DNP = “did not participate.”

### Sampling Strategy and Survey Administration

All HBSC surveys relied on either a one- or a two-stage cluster sample involving a random selection of schools and/or school classes. Questionnaires were self-administered in class.

### Ethics Approval

The HBSC team of every geographic area obtained ethical approval to carry out the survey. The study was conducted in accordance with the principles of the Declaration of Helsinki. Both the participants and their parents provided informed consent.

### Measures

#### Life Satisfaction

LS was assessed with the Cantril’s ladder [30]. Participants were provided with the following description: “Here is a picture of a ladder. The top of the ladder (“10”) [represents] the best possible life for you and the bottom (“0”) [represents] the worst possible life for you.” Participants were invited to indicate where on that ladder they felt they stood (see Supplementary Material 1 and 2 for a summary of this variable’s characteristics).

#### PSS Factors

PSS from family and friends was measured with the corresponding subscales from the Multidimensional Scale of Perceived Social Support [31]. This instrument involves four items assessing family support (e.g., “My family is willing to help me make decisions”; McDonald’s ω = .94) and four items assessing friend support (e.g., “I can count on my friends when things go wrong”; McDonald’s ω = .92). Each item was rated on a 1–7 scale. Mean scores were computed and employed in the conducted analyses (see Supplementary Material 1 for a summary of these variables’ characteristics).

PSS from classmates and teachers was gauged with the corresponding subscales derived from the Teacher and Classmate Support Scale [32] and used in the HBSC study since 2013/14. The measure dedicates three items to classmate support (e.g., “Most of the students in my class(es) are kind and helpful”; McDonald’s ω = .78) and three items to teacher support (e.g., “I feel that my teachers care about me as a person”; McDonald’s ω = .83). Each item was scored on a 1–5 rating scale. We computed and relied on mean scores in the subsequent analyses (see Supplementary Material 1 for a summary of these variables’ characteristics).

#### Sociodemographic Factors

Age was computed based on the month and year of birth and used as a continuous variable. Gender was assessed with a question asking respondents to indicate whether they were a boy or a girl. Family structure was measured with an item asking participants to indicate who they were currently living with based on a predefined list of options (e.g., “father”, “stepfather or mother’s partner”). Participants were allowed to openly specify whether they lived with unlisted individuals (e.g., “grandparents”). We distinguished between intact and non-intact families: respondents reporting living with both their father and mother were assigned to the “intact family” category; the other respondents, to the “non-intact family” category.

SES was assessed with the Family-Affluence-Scale-III (FAS-III) [33]. The FAS-III estimates households’ material living conditions based on six items dedicated to (1) car and (2) dishwasher ownership, (3) the fact of having a personal bedroom, (4) the frequency of holidays abroad, and the number of (5) computers and (6) bathrooms at home. A sum score was then created and used to distinguish between three levels of affluence corresponding to the lowest 20%, the middle 60%, and the highest 20%.

#### Time Factor

Survey year was operationalized as a categorical variable including three modalities: 2013/14, 2017/18, and 2021/22.

#### Additional Control Variable

Because of the well-identified association between mental health and LS [34], we employed a brief inventory developed within the HBSC network [35] to control for mental health issues. The instrument covers the previous 6 months and comprises four items dedicated to low mood, irritability, nervousness, and difficulties in falling asleep. Each item relies on a 1–5 frequency scale ranging from “rarely or never” to “about every day.” A mean score was calculated and used in the performed analyses.

### Statistical Analyses

First, we conducted analyses of variance with pairwise deletion to assess how the level of each source of PSS evolved from 2013/14 to 2021/22. No control variable was used. Tukey *post hoc* tests were carried out to identify potential statistically significant differences at *p* < .05. Since *p* values depend on sample size, and since we relied on relatively large national and regional subsamples, we computed omega squared (ω^2^) to provide effect size estimates. Following Field’s recommendations [36], we distinguished between small (.01 ≤ ω^2^ < .06), moderate (.06 ≤ ω^2^ < .14), and large (ω^2^ ≥ .14) effect sizes. Because Kazakhstan, Russia, Serbia, and Ukraine participated in two waves only, and because a few countries did not systematically employ the whole set of PSS measures, data from these countries were analyzed based on *t*-tests and Cohen’s *d*s. The latter are displayed in Supplementary Material 3. These analyses were carried out with IBM SPSS version 28 (IBM Corp.).

Second, we performed linear mixed modeling analyses to (a) assess the link between LS and our four sources of PSS across space and time and (b) ascertain whether survey year and sociodemographic factors moderated the link in question. In both cases, individuals (level 1) were nested in countries or regions (level 2). We relied on random intercept models. Models including interaction terms were carried out for each source of PSS separately. To further estimate the temporal dynamics of the link between PSS and LS, we reran the analysis split by survey year and with no interaction terms. For ease of interpretation of the unstandardized regression coefficients and to limit multicollinearity, we mean centered our continuous predictors to compute b coefficients and we standardized our continuous variables to compute β coefficients. These analyses were conducted with R, version 4.3.0 (R Foundation for Statistical Computing), using the *lme4* [37] and *performance* [38] packages.

## Results

### Descriptive Statistics and Preliminary Analyses

Participants reported relatively high scores of PSS and LS over the covered period, as indicated by the negatively skewed distribution of these variables (Supplementary Material 1). LS weakly increased between 2013/14 (*M* = 7.64) and 2017/18 (*M* = 7.75) and decreased between 2017/18 and 2021/22 (*M* = 7.44; Supplementary Material 2 and 8).

As shown in Table 2, the largest zero-order correlations involved health complaints and LS (*r* = −.46), family and friend support (*r* = .43), and classmate and teacher support (*r* = .43). PSS from family, classmates, and teachers moderately correlated with LS (.31 ≤ *r*s ≤ .33); PSS from friends exhibited a lower coefficient (*r* = .19). In addition, girls reported lower levels of PSS from family, classmates, and teachers than boys. By contrast, PSS from friends was higher in girls than in boys. Age was negatively correlated with PSS from family, classmates, and teachers. The association between age and PSS from friends was almost null. Adolescents from intact families reported higher levels of PSS than their counterparts, irrespective of the source. SES was positively, albeit weakly correlated with PSS from family, friends, and classmates. The correlation between SES and PSS from teachers was almost null.

**TABLE 2:** Descriptive statistics and zero-order correlations among the study variables (Health Behaviour in School-aged Children study, 44 countries and regions, 2013/14–2021/22).

		*M*	*SD*	2	3	4	5	6	7	8	9	10
1	Family support [1–7]	5.65	1.71	**.43**	.21	.24	−.27	−.12	−.04	.12	.06	**.33**
2	Friend support [1–7]	5.30	1.72	—	.25	.16	−.14	−.01	.09	.06	.06	.19
3	Classmate support [1–5]	3.83	0.85		—	**.43**	**−.30**	−.10	−.07	.09	.05	**.31**
4	Teacher support [1–5]	3.78	0.93			—	**−.30**	−.24	−.03	.08	−.01	**.31**
5	Health complaints [1–5]	2.41	1.09				—	.15	.20	−.10	−.02	**−.46**
6	Age	13.6	1.64					—	.00	−.04	.00	−.18
7	Gender	—	—						—	−.01	.00	−.10
8	Family structure	—	—							—	.13	.14
9	Socioeconomic status	—	—								—	.10
10	Life satisfaction [0–10]	7.60	1.96									—

Note: Coefficients ≤ −.30 or ≥ .30 are bolded.

### Trends in Four Sources of PSS (2013/14–2021/22)

As shown in Table 3, the overall level of each source of PSS slightly lessened between 2013/14 and 2021/22 (all ω^2^ < .010). The decrease in question followed different patterns depending on the source of PSS. PSS from family was rather similar in 2013/14 (*M* = 5.72) and 2017/18 (*M* = 5.69), and then diminished (*M* = 5.57). The same applied to PSS from teachers (*M*_2013/14_ = 3.84; *M*_2017/18_ = 3.81; *M*_2021/22_ = 3.71). The decrease in PSS from friends mainly occurred between 2014 and 2018 (*M*_2013/14_ = 5.42; *M*_2017/18_ = 5.27; *M*_2021/22_ = 5.23). Finally, the decrement in PSS from classmates was relatively constant over the examined period (*M*_2013/14_ = 3.91; *M*_2017/18_ = 3.84; *M*_2021/22_ = 3.74).

**TABLE 3:** Trends in the mean scores pertaining to four sources of perceived social support (Health Behaviour in School-aged Children study, 44 countries and regions, 2013/14–2021/22).

	Family support [1–7]				Friend support [1–7]				Classmate support [1–5]				Teacher support [1–5]			
	2014	2018	2022	ω^2^	2014	2018	2022	ω^2^	2014	2018	2022	ω^2^	2014	2018	2022	ω^2^
Albania	6.30^ab^	6.37^a^	6.26^b^	.001	5.75^a^	5.48^b^	5.65^c^	.003	4.32^a^	4.23^b^	4.25^b^	.003	4.32^a^	4.30^a^	4.33^a^	.000
Armenia	5.93^a^	5.91^a^	5.79^b^	.001	–	5.52^a^	5.51^a^	–	4.25^a^	4.33^b^	4.10^c^	**.015**	4.13^a^	4.10^a^	3.93^b^	**.010**
Austria	6.00^a^	5.88^b^	5.90^b^	.001	5.64^a^	5.68^a^	5.64^a^	.000	4.08^a^	4.02^b^	3.96^c^	.004	3.89^a^	3.76^b^	3.58^c^	**.017**
Bulgaria	5.08^a^	4.57^b^	5.07^a^	**.014**	4.91^a^	4.55^b^	4.85^a^	.007	3.55^a^	3.62^b^	3.44^c^	.005	3.73^a^	3.71^a^	3.52^b^	.008
Canada	–	4.98^a^	4.92^b^	–	–	4.81^a^	4.70^b^	–	3.65^a^	3.60^b^	3.48^c^	.007	3.82^a^	3.86^b^	3.81^a^	.001
Croatia	6.08^a^	6.08^a^	5.84^b^	.005	5.76^a^	5.52^b^	5.47^b^	.006	3.96^a^	3.84^b^	3.87^b^	.004	3.84^a^	3.66^b^	3.73^c^	.005
Czechia	6.03^a^	5.05^b^	5.55^c^	**.035**	5.50^a^	4.64^b^	5.15^c^	**.032**	3.59^a^	3.56^a^	3.39^b^	**.011**	3.49^a^	3.58^b^	3.41^c^	.007
Denmark	–	–	6.08	–	–	5.99^a^	5.70^b^	–	4.09^a^	4.04^b^	3.93^c^	.008	4.06^a^	4.03^ab^	4.00^b^	.001
England	5.43^a^	4.92^b^	4.98^b^	**.015**	5.23^a^	4.50^b^	4.91^c^	**.025**	3.86^a^	3.65^b^	3.49^c^	**.037**	3.96^a^	3.77^b^	3.55^c^	**.036**
Estonia	5.59^a^	5.93^b^	5.75^c^	.008	5.02^a^	5.30^b^	5.33^b^	.006	3.83^a^	3.83^a^	3.76^b^	.002	3.69^a^	3.72^ab^	3.75^b^	.001
Finland	5.81^a^	5.69^b^	5.61^b^	.002	5.57^a^	5.45^b^	5.47^b^	.001	3.88^a^	3.90^a^	3.79^b^	.003	3.70^a^	3.85^b^	3.96^c^	**.014**
Flanders	5.41^a^	5.87^b^	5.69^c^	**.010**	5.23^a^	5.60^b^	5.33^c^	.008	3.96^a^	4.05^b^	3.93^a^	.004	3.81^a^	3.97^b^	3.76^c^	.008
France	5.75^a^	5.83^b^	5.59^c^	.004	5.73^a^	5.48^b^	5.55^b^	.004	3.76^a^	3.86^b^	3.66^c^	.008	3.63^a^	3.81^b^	3.62^a^	**.010**
Germany	5.76^a^	5.84^b^	5.61^c^	.004	5.57^a^	5.61^a^	5.49^b^	.001	4.08^a^	4.07^a^	3.92^b^	**.010**	3.81^a^	3.75^b^	3.57^c^	**.015**
Greece	6.08^a^	6.01^a^	5.82^b^	.006	5.84^a^	5.72^b^	5.55^c^	.007	3.68^a^	3.62^b^	3.58^b^	.002	3.88^a^	3.74^b^	3.65^c^	**.010**
Greenland	4.80^a^	5.37^b^	4.46^c^	**.047**	4.68^a^	5.20^b^	4.75^a^	**.018**	3.79^a^	3.90^b^	3.85^ab^	.002	4.01^a^	4.10^ab^	4.11^b^	.002
Hungary	6.15^a^	6.20^a^	6.00^b^	.005	5.97^a^	6.01^a^	5.78^b^	.005	3.88^a^	3.76^b^	3.60^c^	**.016**	3.76^a^	3.64^b^	3.50^c^	**.012**
Iceland	5.07^a^	5.64^b^	5.73^c^	.**022**	4.90^a^	5.31^b^	5.11^c^	.007	4.06^a^	3.97^b^	3.75^c^	**.030**	4.07^a^	4.03^b^	3.91^c^	.007
Ireland	5.16^a^	5.30^b^	5.16^a^	.001	5.12^a^	5.25^b^	5.24^b^	.001	3.97^a^	3.96^a^	3.87^b^	.003	3.75^a^	3.85^b^	3.78^a^	.002
Israel	5.92^a^	5.92^a^	5.81^b^	.001	5.35^a^	5.27^b^	5.34^a^	.000	4.02^a^	3.94^b^	3.77^c^	**.013**	3.90^a^	3.98^b^	3.70^c^	**.016**
Italy	6.05^a^	5.96^b^	5.53^c^	**.023**	5.82^a^	5.62^b^	5.45^c^	.009	3.95^a^	3.93^a^	3.84^b^	.003	3.74^a^	3.75^a^	3.61^b^	.005
Kazakhstan	–	6.02^a^	5.82^b^	–	–	5.28^a^	5.39^b^	–	–	3.98^a^	3.90^b^	–	–	4.12^a^	3.91^b^	–
Latvia	5.15^a^	5.55^b^	5.20^a^	.008	4.71^a^	4.89^b^	4.88^b^	.002	3.69^a^	–	3.54^b^	–	3.79^a^	–	3.61^b^	–
Lithuania	–	5.79^a^	5.32^b^	–	5.37^a^	5.23^b^	5.22^b^	.002	3.68^a^	3.64^ab^	3.61^b^	.001	3.77^a^	3.62^b^	3.38^c^	**.032**
Luxembourg	5.32^a^	5.82^b^	5.56^c^	**.014**	5.34^a^	5.61^b^	5.42^a^	.005	3.99^a^	3.96^a^	3.84^b^	.007	3.80^a^	3.67^b^	3.64^b^	.005
Macedonia	6.17^a^	6.50^b^	6.01^c^	**.022**	5.54^a^	5.77^b^	–	–	4.22^a^	4.20^a^	4.19^a^	.000	4.15^a^	4.09^b^	3.99^c^	.005
Malta	5.76^a^	5.91^b^	5.68^a^	.004	5.36^a^	5.56^b^	5.47^c^	.002	4.00^a^	3.98^ab^	3.94^b^	.001	4.06^a^	4.00^b^	3.92^c^	.004
Moldova	5.94^a^	5.76^b^	5.47^c^	**.011**	5.27^a^	4.94^b^	4.75^c^	**.012**	4.04^a^	3.88^b^	3.77^c^	**.018**	3.92^a^	3.89^a^	3.63^b^	**.022**
Netherlands	5.94^a^	6.12^b^	5.92^a^	.005	5.75^a^	5.79^a^	5.59^b^	.004	4.13^a^	4.07^b^	4.02^c^	.004	3.92^a^	3.87^b^	3.91^ab^	.001
Norway	6.15^a^	6.23^b^	5.94^c^	**.010**	5.66^a^	5.71^a^	5.33^b^	**.012**	4.22^a^	4.18^a^	3.96^b^	**.022**	4.15^a^	4.15^a^	4.08^b^	.001
Poland	5.56^a^	5.52^a^	5.04^b^	**.021**	5.16^a^	4.46^b^	4.40^b^	**.040**	3.82^a^	–	3.51^b^	–	3.65^a^	–	3.28^b^	–
Portugal	5.82^a^	6.07^b^	5.79^a^	.006	5.56^a^	5.51^ab^	5.45^b^	.001	4.02^a^	3.97^b^	3.94^c^	.002	3.87^a^	3.81^b^	3.81^b^	.001
Romania	6.08^a^	6.08^a^	5.75^b^	**.010**	5.36^a^	5.22^b^	5.17^b^	.002	4.02^a^	3.93^b^	3.73^c^	**.021**	3.84^a^	3.75^b^	3.57^c^	**.014**
Russia	5.31^a^	5.53^b^	–	–	4.84^a^	4.62^b^	–	–	3.64^a^	3.59^b^	–	–	3.60^a^	3.50^b^	–	–
Scotland	5.51^a^	5.23^b^	5.23^b^	.005	5.36^a^	5.04^b^	5.06^b^	.007	3.71^a^	3.64^b^	3.42^c^	**.020**	3.66^a^	3.90^b^	3.66^a^	**.014**
Serbia	–	6.31^a^	6.01^b^	–	–	5.38^a^	5.53^b^	–	–	3.89^a^	3.95^b^	–	–	3.53^a^	3.53^a^	–
Slovakia	6.09^a^	5.79^b^	5.58^c^	**.021**	5.57^a^	5.15^b^	5.12^b^	**.017**	3.73^a^	3.66^b^	3.68^b^	.001	3.57^ab^	3.58^a^	3.53^b^	.000
Slovenia	5.66^a^	5.14^b^	5.51^c^	**.011**	5.34^a^	5.15^b^	5.27^a^	.002	4.19^a^	4.03^b^	3.89^c^	**.022**	3.82^a^	3.74^b^	3.64^c^	.006
Spain	5.95^a^	6.09^b^	5.67^c^	.009	5.68^a^	6.03^b^	5.59^c^	**.012**	3.97^a^	3.94^a^	3.84^b^	.003	3.78^a^	3.73^b^	3.61^c^	.004
Sweden	5.76^a^	6.05^b^	6.02^b^	**.011**	5.65^a^	5.66^a^	5.54^b^	.001	4.13^a^	3.92^b^	3.73^c^	**.045**	4.27^a^	4.10^b^	3.99^c^	**.020**
Switzerland	6.01^a^	5.97^a^	6.10^b^	.002	5.87^a^	5.67^b^	5.77^c^	.005	4.08^a^	3.99^b^	3.98^b^	.003	3.90^ab^	3.87^a^	3.91^b^	.000
Ukraine	5.45^a^	5.65^b^	–	–	5.00^a^	4.86^b^	–	–	3.75^a^	3.50^b^	–	–	3.67^a^	3.54^b^	–	–
Wales	5.25^a^	5.30^a^	5.16^b^	.001	5.08^a^	5.00^a^	4.89^b^	.001	3.73^a^	3.60^b^	3.54^c^	.005	3.68^a^	3.68^a^	3.61^b^	.001
Wallonia	5.52^a^	5.84^b^	5.46^a^	**.010**	5.68^a^	5.48^b^	5.23^c^	**.014**	3.87^a^	3.91^a^	3.77^b^	.005	3.77^a^	3.87^b^	3.75^a^	.003
Sample	5.72^a^	5.69^b^	5.57^c^	.001	5.42^a^	5.27^b^	5.23^c^	.002	3.91^a^	3.84^b^	3.74^c^	.007	3.84^a^	3.81^b^	3.71^c^	.004

Notes: When differing horizontally within a given source of perceived social support, superscript letters signal a statistically significant difference identified based on Tukey *post hoc* tests (when data pertaining to all three waves were available) or on *t*-test (when data pertaining to two waves only were available). Omega squared ≥ .010 are bolded.

This general description masks heterogeneous local dynamics (Table 3; Supplementary Material 4–7). Although the comprehensive assessment of these dynamics is beyond the scope of this study, three points are worth noticing. First, while PSS from each source diminished in most countries and regions between 2013/14 and 2021/22, both the temporal patterns and the magnitude of this decline varied with national context and sources of PSS. For instance, PSS from family steadily decreased in Italy, Moldova, and Slovakia; PSS from classmates, in 15 countries, with ω^2^ ranging from .002 in Portugal to .045 in Sweden. Second, we pinpointed no clear geographical or cultural pattern accounting for the variability of the temporal dynamics reported in Table 3. Third, most ω^2^ were negligible. PSS from family involved 17 small ω^2^ out of 37 effect size estimates; PSS from friends, 9 out of 36; PSS from classmates, 13 out of 38; PSS from teachers, 14 out of 38. We identified no medium or large effect size (Table 3).

### Associations Between Sources of PSS and LS Across Space and Time

Our linear mixed model revealed that each source of PSS was positively associated with LS (Table 4). PSS from family involved the largest association (β = 0.16) and PSS from friends, the smallest one (β = 0.03). Given its small magnitude, the latter association can be considered of minor importance. PSS from classmates and teachers related to LS to a similar extent (both βs = 0.11).

**TABLE 4:** Summary of linear mixed modeling analysis predicting life satisfaction (Health Behaviour in School-aged Children study, 44 countries and regions, 2013/14–2021/22).

*Fixed effects*	b	β	SE	*t*-value	*p*-value	95% CI
Family support [1–7]	0.18	0.16	0.001	116.74	<.001	0.15, 0.16
Friend support [1–7]	0.03	0.03	0.001	21.61	<.001	0.03, 0.03
Classmate support [1–5]	0.25	0.11	0.001	80.15	<.001	0.10, 0.11
Teacher support [1–5]	0.23	0.11	0.001	80.58	<.001	0.11, 0.11
Health complaints [1–5]	−0.57	−0.32	0.001	−245.42	<.001	−0.32, −0.32
Age	−0.09	−0.08	0.001	−65.92	<.001	−0.08, −0.08
Gender						
Girls vs. boys	−0.06	−0.03	0.002	−14.02	<.001	−0.04, −0.03
Family structure						
Intact vs. non-intact	0.25	0.13	0.003	47.53	<.001	0.12, 0.13
Socioeconomic status						
Medium vs. low	0.25	0.13	0.003	42.77	<.001	0.12, 0.13
High vs. low	0.48	0.24	0.004	66.74	<.001	0.24, 0.25
Survey year						
2014 vs. 2018	−0.19	−0.10	0.003	−32.70	<.001	−0.10, −0.09
2022 vs. 2018	−0.03	−0.02	0.003	−5.89	<.001	−0.02, −0.01
***Random effect (intercept)***	**σ^2^**	***SD***				
Country or region	0.02	0.12				
***Model statistics***						
AIC	1,214,581					
BIC	1,214,748					
Marginal *R* ^2^	.31					
Conditional *R* ^2^	.32					
Intraclass correlation	.02					
No. of individuals	505,848					
No. of countries/regions	44					

Notes: Continuous predictors were mean centered to compute b coefficients. Continuous variables were standardized to compute β coefficients. No multicollinearity issue was detected: all variation inflation factors were < 1.4.

The country-and-region level accounted for a marginal amount of variance in LS (intraclass correlation = .02), suggesting that the relationship between our predictors and LS varied with national or regional context to a negligible extent (Table 4). We also found LS level to be lower in 2013/14 than in 2017/18 (β = −0.10) and to be rather similar in 2017/18 and 2021/22 (β = −0.02).

As shown in Table 5, survey year weakly moderated the link between LS and each source of PSS (−0.01 ≤ βs ≤ 0.06). Rerunning the same analysis split by survey year (Table 6) indicated that the association between PSS from family and LS was similar in 2013/14 (β = 0.14) and 2017/18 (β = 0.15) and slightly increased afterward (β = 0.18). PSS from friends showed negligible, albeit growing associations with LS over time (0.02 ≤ βs ≤ 0.04). While the association between PSS from classmates and LS steadily weakened (β_2013/14_ = 0.13; β_2017/18_ = 0.11; β_2021/22_ = 0.09), the one between PSS from teachers and LS was rather stable over time (β_2013/14_ = 0.12; β_2017/18_ = 0.10: β_2021/22_ = 0.11).

**TABLE 5:** Summary of linear mixed modeling analyses with interaction terms predicting life satisfaction, source of social support per source of social support (Health Behaviour in School-aged Children study, 44 countries and regions, 2013/14–2021/22).

**Fixed effects**	Model 1: Perceived family support as “interactor”					Model 2: Perceived friend support as “interactor”				
	b	β	SE	*p*	95% CI	b	β	SE	*p*	95% CI
Family support [1–7]	0.17	0.14	0.004	<.001	0.14, 0.15	0.18	0.16	0.001	<.001	0.15, 0.16
Friend support [1–7]	0.03	0.03	0.001	<.001	0.03, 0.03	0.04	0.03	0.004	<.001	0.02, 0.04
Classmate support [1–5]	0.24	0.11	0.001	<.001	0.10, 0.11	0.25	0.11	0.001	<.001	0.10, 0.11
Teacher support [1–5]	0.22	0.11	0.001	<.001	0.10, 0.11	0.23	0.11	0.001	<.001	0.11, 0.11
Health complaints [1–5]	−0.56	−0.32	0.001	<.001	−0.32, −0.31	−0.57	−0.32	0.001	<.001	−0.32, −0.32
Age	−0.09	−0.08	0.001	<.001	−0.08, −0.08	−0.09	−0.08	0.001	<.001	−0.08, −0.08
Gender										
Girls vs. boys	−0.07	−0.03	0.002	<.001	−0.04, −0.03	−0.06	−0.03	0.002	<.001	−0.04, −0.03
Family structure										
Intact vs. non-intact	0.24	0.12	0.003	<.001	0.12, 0.13	0.25	0.13	0.003	<.001	0.12, 0.13
Socioeconomic status										
Medium vs. low	0.24	0.12	0.003	<.001	0.12, 0.13	0.25	0.13	0.003	<.001	0.12, 0.13
High vs. low	0.47	0.24	0.004	<.001	0.24, 0.25	0.48	0.24	0.004	<.001	0.24, 0.25
Survey year										
2014 vs. 2018	−0.19	−0.10	0.003	<.001	−0.10, −0.09	−0.19	−0.10	0.003	<.001	−0.10, −0.09
2022 vs. 2018	−0.03	−0.02	0.003	<.001	−0.02, −0.01	−0.03	−0.02	0.003	<.001	−0.02, −0.01
*Age	0.01	0.01	0.001	<.001	0.01, 0.01	0.00	0.00	0.001	.434	0.00, 0.00
*Gender										
Girls vs. boys	0.07	0.06	0.002	<.001	0.06, 0.07	0.01	0.01	0.002	<.001	0.01, 0.02
*Family structure										
Intact vs. non-intact	−0.02	−0.02	0.003	<.001	−0.03, −0.02	0.00	0.00	0.003	.423	0.00, 0.01
*Socioeconomic status										
Medium vs. low	−0.03	−0.03	0.003	<.001	−0.04, −0.02	−0.02	−0.02	0.003	<.001	−0.03, −0.02
High vs. low	−0.06	−0.06	0.004	<.001	−0.06, −0.05	−0.05	−0.04	0.004	<.001	−0.05, −0.03
*Survey year										
2014 vs. 2018	0.00	0.00	0.003	.521	0.00, 0.01	−0.01	−0.01	0.003	.021	−0.01, 0.00
2022 vs. 2018	0.06	0.06	0.003	<.001	0.05, 0.06	0.03	0.03	0.003	<.001	0.03, 0.04
***Random effect (intercept)***	**σ^2^**	***SD***				**σ^2^**	***SD***			
Country or region	0.01	0.12				0.01	0.12			
***Model statistics***										
AIC	1,212,900					1,214,311				
BIC	1,213,145					1,214,556				
Marginal *R* ^2^	.31					.31				
Conditional *R* ^2^	.33					.32				

Fixed effects	Model 3: Perceived classmate support as “interactor”					Model 4: Perceived teacher support as “interactor”				
	b	β	SE	*p*	95% CI	b	β	SE	*p*	95% CI
Family support [1–7]	0.18	0.15	0.001	<.001	0.15, 0.16	0.18	0.15	0.001	<.001	0.15, 0.16
Friend support [1–7]	0.03	0.03	0.001	<.001	0.03, 0.03	0.03	0.03	0.001	<.001	0.03, 0.03
Classmate support [1–5]	0.26	0.11	0.004	<.001	0.11, 0.12	0.24	0.11	0.001	<.001	0.10, 0.11
Teacher support [1–5]	0.23	0.11	0.001	<.001	0.11, 0.11	0.20	0.10	0.004	<.001	0.09, 0.10
Health complaints [1–5]	−0.57	−0.32	0.001	<.001	−0.32, −0.32	−0.57	−0.32	0.001	<.001	−0.32, −0.32
Age	−0.09	−0.08	0.001	<.001	−0.08, −0.07	−0.09	−0.08	0.001	<.001	−0.08, −0.07
Gender										
Girls vs. boys	−0.07	−0.03	0.002	<.001	−0.04, −0.03	−0.07	−0.03	0.002	<.001	−0.04, −0.03
Family structure										
Intact vs. non-intact	0.24	0.12	0.003	<.001	0.12, 0.13	0.25	0.13	0.003	<.001	0.12, 0.13
Socioeconomic status										
Medium vs. low	0.25	0.13	0.003	<.001	0.12, 0.13	0.25	0.13	0.003	<.001	0.12, 0.13
High vs. low	0.48	0.24	0.004	<.001	0.24, 0.25	0.48	0.24	0.004	<.001	0.24, 0.25
Survey year										
2014 vs. 2018	−0.20	−0.10	0.003	<.001	−0.11, −0.09	−0.19	−0.10	0.003	<.001	−0.10, −0.09
2022 vs. 2018	−0.03	−0.02	0.003	<.001	−0.02, −0.01	−0.03	−0.02	0.003	<.001	−0.02, −0.01
*Age	−0.01	−0.01	0.001	<.001	−0.01, −0.01	−0.01	−0.01	0.001	<.001	−0.01, −0.01
*Gender										
Girls vs. boys	0.05	0.02	0.002	<.001	0.02, 0.02	0.09	0.04	0.002	<.001	0.04, 0.05
*Family structure										
Intact vs. non-intact	−0.02	−0.01	0.003	.002	−0.01, 0.00	−0.01	0.00	0.003	.305	−0.01, 0.00
*Socioeconomic status										
Medium vs. low	−0.06	−0.03	0.003	<.001	−0.03, −0.02	−0.05	−0.02	0.003	<.001	−0.03, −0.02
High vs. low	−0.11	−0.05	0.004	<.001	−0.05, −0.04	−0.10	−0.05	0.004	<.001	−0.05, −0.04
*Survey year										
2014 vs. 2018	0.08	0.03	0.003	<.001	0.03, 0.04	0.07	0.03	0.003	<.001	0.03, 0.04
2022 vs. 2018	0.03	0.01	0.003	<.001	0.01, 0.02	0.05	0.02	0.003	<.001	0.02, 0.03
***Random effect (intercept)***	**σ^2^**	***SD***				**σ^2^**	***SD***			
Country or region	0.01	0.12				0.01	0.12			
***Model statistics***										
AIC	1,214,218					1,213,941				
BIC	1,214,463					1,214,186				
Marginal *R* ^2^	.31					.31				
Conditional *R* ^2^	.32					.32				

Notes: The symbol * indicates an interaction term. Each model involved 505,048 individuals, 44 countries and regions, and an intraclass correlation of .02.

**TABLE 6:** Summary of linear mixed modeling analysis predicting life satisfaction, split by survey year (Health Behaviour in School-aged Children study, 44 countries and regions, 2013/14–2021/22).

*Fixed effects*	2013/14				2017/18				2021/22			
	b [95% CI]		SE	β	b [95% CI]		SE	β	b [95% CI]		SE	β
Family support [1–7]	0.16 [0.16, 0.17]		0.003	0.14	0.16 [0.16, 0.17]		0.003	0.15	0.21 [0.20, 0.21]		0.002	0.18
Friend support [1–7]	0.02 [0.01, 0.03]		0.003	0.02	0.03 [0.03, 0.04]		0.002	0.03	0.04 [0.04, 0.05]		0.002	0.04
Classmate support [1–5]	0.30 [0.29, 0.31]		0.006	0.13	0.24 [0.23, 0.25]		0.005	0.11	0.21 [0.20, 0.22]		0.005	0.09
Teacher support [1–5]	0.26 [0.24, 0.27]		0.006	0.12	0.21 [0.20, 0.22]		0.005	0.10	0.22 [0.21, 0.23]		0.004	0.11
Health complaints [1–5]	−0.54 [−0.55, −0.53]		0.005	−0.29	−0.57 [−0.57, −0.56]		0.004	−0.31	−0.60 [−0.61, −0.59]		0.004	−0.34
Age	−0.09 [−0.10, −0.09]		0.003	−0.08	−0.09 [−0.10, −0.09]		0.002	−0.08	−0.10 [−0.10, −0.09]		0.002	−0.08
Gender												
Girls vs. boys	−0.08 [−0.10, −0.06]		0.009	−0.04	−0.04 [−0.05, −0.02]		0.008	−0.02	−0.07 [−0.09, −0.06]		0.007	−0.04
Family structure												
Intact vs. non-intact	0.27 [0.25, 0.29]		0.010	0.14	0.26 [0.24; 0.27]		0.009	0.13	0.22 [0.20, 0.23]		0.008	0.11
Socioeconomic status												
Medium vs. low	0.26 [0.23, 0.28]		0.011	0.13	0.28 [0.26, 0.30]		0.010	0.15	0.22 [0.197, 0.233]		0.009	0.11
High vs. low	0.50 [0.47, 0.52]		0.014	0.26	0.53 [0.51, 0.56]		0.012	0.28	0.43 [0.408, 0.452]		0.011	0.22
***Random effect (intercept)***	**σ^2^**	***SD***			**σ^2^**	***SD***			**σ^2^**	***SD***		
Country or region	0.02	0.13			0.02	0.12			0.03	0.16		
***Model statistics***												
Marginal *R* ^2^	.26				.28				.36			
Conditional *R* ^2^	.28				.30				.39			
Intraclass correlation	.02				.02				.04			

Notes: Continuous predictors were mean centered to compute b coefficients. Continuous variables were standardized to compute β coefficients. All *p* values were <.001. No multicollinearity issue was detected: all variation inflation factors were < 1.4.

### The Moderating Role of Sociodemographic Factors

Age did not affect the link between PSS from friends and LS (β = 0.00) and negligibly moderated the relationship between LS and the other sources of PSS (−0.01 ≤ βs ≤ 0.01 [Table 5]).

LS levels were rather similar in girls and in boys (β = −0.03) within our “main effect” model (Table 4). As shown in Table 5, gender weakly moderated the link between PSS from family and LS (β = 0.06) and between PSS from teachers and LS (β = 0.04). In both cases, the higher the level of PSS, the smaller the gender gap in LS. Gender negligibly moderated the link between PSS from friends and LS (β = 0.01) and between PSS from classmates and LS (β = 0.02).

Family structure did not affect the association between PSS from friends and LS and between PSS from teachers and LS (both βs = 0.00). Although the interaction coefficients involving PSS from family (β = −0.02) and classmates (β = −0.01) reached statistical significance, their small magnitude suggests a negligible moderating role (Table 5).

LS levels appeared positively associated with SES within our “main effect” model (Table 4). As shown in Table 5, SES slightly moderated the link between PSS and LS, irrespective of the source (−0.06 ≤ βs ≤ −0.02): the higher the PSS, the smaller the LS gap between SES.

## Discussion

The present study aimed to (a) report temporal trends in four sources of PSS, (b) assess the link between PSS and LS across space and time, and (c) estimate whether age, family structure, gender, and SES moderated this link.

### Trends in PSS (2013/14–2021/22)

Overall, our findings highlighted a small decrement in each source of PSS between 2013/14 and 2021/22. PSS from classmates involved the largest, the most widespread, and the steadiest decrease; PSS from family, the smallest one. While a decline in PSS occurred in most geographic areas, this decline was not pervasive and both its pace and magnitude varied with national and regional context. Despite these downward trends, the average level of the examined sources of PSS remained relatively high over time. This finding is in line with past research. In effect, studies relying on representative samples have reported similar results in that respect, both in adults and non-adults [28, 39].

The dearth of studies examining trends in PSS prevents us from connecting our findings to a structured body of literature. One of the rare diachronic analyses available [28] found adolescents’ PSS to substantially increase between 2011 and 2012 and to slightly decrease between 2014 and 2018 in Canada. While this finding partly corroborates ours, it should be noted that this Canadian study relied on a generic measure of PSS, which limits comparison with our results.

### The Link Between PSS and LS Across Space and Time

We found the association between our predictors and LS to weakly vary with national or regional context. This result is in keeping with previous research. As an illustration, a study conducted in Israel and Singapore highlighted no national difference in the link between LS and any of the five sources of PSS examined [21]. In their study of 42 geographic areas, Bi et al. [13] emphasized national differences in the extent to which distinct sources of PSS related to LS. However, the intraclass correlation coefficients pertaining to the national level reported by these authors were similar to ours. This being said, (sub)cultural variability in parental style [40], social perception of help-seeking [41], and victimization prevalence [42] may narrow or neutralize the positive relationship between a given source of PSS and LS in certain national contexts. In addition, LS assessment may involve different criteria depending on cultural milieu, geographic area, and social contingency. For example, an adolescent may report a high level of PSS from teachers and a low level of LS based on criteria extraneous to the school environment (e.g., romantic disappointment, parent’s job loss). Future studies may address these issues.

All waves considered, PSS from the family involved the largest association with LS. This general result veils subtle differences in the temporal dynamics of the positive link between PSS and LS, though. PSS from family, classmates, and teachers related to a rather similar extent to LS in 2013/14 and 2017/18. The situation changed in 2021/22, with perceived family support exhibiting the largest association with LS. The COVID-19 pandemic and the inflation rise intervening since 2021 in Europe [43] may have strengthened the interdependence of family support and LS. For instance, the restrictive measures implemented during the pandemic may have exacerbated family conflicts and/or (re)invigorated family ties [44, 45], two situations that bear on the link between family support and LS. Inflation and post-pandemic economic adjustments may have impacted the instrumental support provided to adolescents, primarily involving parental assistance [46, 47]. Be that as it may, our results partly echo those of meta-analyses. Rueger et al. [15] identified family and peers as the sources of PSS predicting depression to the largest extent. Although relying on a questionable operationalization of wellbeing (e.g., including academic performance), Chu et al. [10] pinpointed teachers and family as the sources of PSS involving the greatest associations with wellbeing.

All in all, our study suggests that the levels of PSS and the associations between sources of PSS and LS subtly evolve and may require to be examined in the context of macrosocial phenomena. The case of PSS from classmates is emblematic in that respect: both its general level and its degree of association with LS were indeed the only ones to steadily diminish since 2013/14. The extent to which such a decline is due to the crescent digitalization of social life is unclear. Although one may claim, based on the literature [48], that classmates have been gradually supplanted by (digital) peers outside the attended school in youths’ rewarding environment and popularity systems, this research area is only burgeoning [49]. Similarly, the extent to which adolescents rely on digital tools to get informational support is unclear. Further research is needed to identify the macrosocial dynamics that may affect PSS and its positive association with LS.

### The Moderating Role of Age, Family Structure, Gender, and SES

The analyses conducted with no interaction terms revealed a negative association between age and LS and a positive relationship between SES and LS. They also revealed gender differences, with girls reporting slightly less LS than boys. Furthermore, these analyses indicated that living with both parents was linked to higher LS levels. Overall, these results do not align with those reported in early studies, which emphasized marginal associations between sociodemographic factors and LS [50, 51], and corroborate those emanating from recent research [52, 53].

By contrast, the analyses including interaction terms showed that both age and family structure negligibly moderated the association between PSS and LS. Gender was found to slightly affect the link between perceived family support and LS and between perceived teacher support and LS only. Girls’ LS appeared to depend on PSS from adults to a larger extent than boys’ LS. The extent to which this disparity stems from (a) sex differences in puberty and its correlates (e.g., in terms of internalizing disorders) [52, 54], (b) personality differences (e.g., in terms of neuroticism, self-esteem, and self-efficacy), [55–57], and/or (c) gendered coping strategies [58] remains open to question. Finally, SES slightly moderated the association between LS and each source of PSS, with higher levels of PSS mitigating LS gaps between SES. One possible explanation of this result relies on the scarcity principle: the lower availability of PSS in lower SES may increase its weight on LS assessment within this status group. Future studies may address this under-investigated issue in adolescents.

### General Considerations

Our study illustrates the relevance of distinguishing between general peers (here, classmates) and friends when estimating the link between PSS and wellbeing or health outcomes [15]. While PSS from classmates showed *relatively* high—albeit decreasing over time—associations with LS, PSS from friends was negligibly linked to LS. Several hypotheses have been formulated to account for the weakness of that link, from the ephemerality and instability of adolescents’ friendships [22] to co-rumination [15]. Unfortunately, these hypotheses have not been tested to date. Another potential explanation lies in the “dark side” of friend support that may encourage adolescents to engage in risky or deviant behaviors [59–61]. Such behaviors have been linked to lower levels of LS [62, 63]. However, because our correlation analyses were indicative of a positive association between perceived friend support and LS, it should not be excluded that the limited predictive power of the former on the latter might be the consequence of a suppression effect. Future studies may attempt to identify the suppressor(s) in question and clarify the role of classmate (or general peer) support.

More generally, further research is needed to pinpoint the principles underlying the link between each source of PSS and LS. Building on the observation that adolescents expect and/or seek specific types of support from specific categories of individuals [46, 47, 64], the examination of (a) the interplay of sources of PSS, types of PSS, and dimensions of LS and (b) potential compensatory effects between sources of PSS [19] may help delineate these principles. Such studies may allow investigators to better map the action range of each source of PSS and enhance the understanding of the associations between sources of PSS and LS. In addition, because PSS and LS have shown moderate-to-large associations with the five-factor model of personality, self-esteem, and social skills [9, 65–68], considering these factors may represent an added value in the theorization of the relationship between PSS and LS.

### Limitations and Strengths

At least three limitations to the present study can be noted. First, the cross-sectional nature of our work did not allow us to assess potential bidirectional relationships between PSS and LS and to draw causal inferences. For instance, it should not be excluded that lower LS may undermine social interactions, the maintenance of good relationships, and the building of new rewarding relationships. Second, we relied on self-reported data, which involve well-identified biases (e.g., social desirability, response style). Third, our measures of PSS stemmed from two distinct inventories that may capture slightly different conceptualizations of PSS. While the scales assessing PSS from family and friends primarily deal with general support, those dedicated to PSS from classmates and teachers primarily deal with emotional support and classroom climate. Because there is evidence that adolescents solicit different providers depending on the type of social provision they seek, using the same items to estimate PSS from different sources may be unwarranted. However, future research is needed to (a) further establish that the measures of PSS from classmates and teachers used in the HBSC survey have to do with PSS only and (b) pinpoint the specific support provided by distinct sources (including digital ones).

At least three strengths of our work can be highlighted. First, our study contributed to filling a critical gap in the literature, namely, the lack of studies examining the temporal dynamics of PSS. Second, the HBSC study involves a standardized research protocol that ensures high levels of consistency across space and time (e.g., in terms of survey instruments, data collection and processing procedures). Third, we relied on a large sample consisting of nationally and regionally representative subsamples of school attendees. This permitted us to report generalizable estimates and to provide investigators with reliable points of comparison for future research.
