# Supplementary material for: Trends in the Link Between Perceived Social Support and Life Satisfaction in Adolescents (2013/14–2021/22): A Cross-National Study
Source: Int J Public Health. 2024 Jul 10;69:1607283. doi: 10.3389/ijph.2024.1607283 (PMC11266036; doi:10.3389/ijph.2024.1607283)

| **Supplementary Material 1.** Descriptive characteristics of the four sources of perceived social support, health complaints, and life satisfaction (*Health Behaviour of School-aged Children*, 44 countries and regions, 2013/14–2021/22). | | | | | | | | | | | | | | | |
| --- | --- | --- | --- | --- | --- | --- | --- | --- | --- | --- | --- | --- | --- | --- | --- |
|  | **2014** | | |  | **2018** | | |  | **2022** | | |  | **2014-2022** | | |
|  | *Mean* | *Median* | *SD* |  | *Mean* | *Median* | *SD* |  | *Mean* | *Median* | *SD* |  | *Mean* | *Median* | *SD* |
| Family support | 5.72 | 6.50 | 1.68 |  | 5.69 | 6.50 | 1.73 |  | 5.57 | 6.25 | 1.72 |  | 5.65 | 6.25 | 1.71 |
| Friend support | 5.42 | 6.00 | 1.69 |  | 5.27 | 5.75 | 1.75 |  | 5.23 | 5.75 | 1.71 |  | 5.30 | 5.75 | 1.72 |
| Classmate support | 3.91 | 4.00 | 0.82 |  | 3.84 | 4.00 | 0.85 |  | 3.74 | 4.00 | 0.87 |  | 3.83 | 4.00 | 0.85 |
| Teacher support | 3.84 | 4.00 | 0.91 |  | 3.81 | 4.00 | 0.93 |  | 3.71 | 3.67 | 0.96 |  | 3.78 | 4.00 | 0.93 |
| Health complaints | 2.21 | 2.00 | 1.03 |  | 2.33 | 2.25 | 1.04 |  | 2.62 | 2.50 | 1.14 |  | 2.41 | 2.25 | 1.09 |
| Life satisfaction | 7.64 | 8.00 | 1.95 |  | 7.75 | 8.00 | 1.91 |  | 7.44 | 8.00 | 2.00 |  | 7.60 | 8.00 | 1.96 |
|  | *Mode* | *Skew.* | *Kurt.* |  | *Mode* | *Skew.* | *Kurt.* |  | *Mode* | *Skew.* | *Kurt.* |  | *Mode* | *Skew.* | *Kurt.* |
| Family support | 7.00 | -1.51 | 1.37 |  | 7.00 | -1.43 | 1.05 |  | 7.00 | -1.20 | 0.42 |  | 7.00 | -1.36 | 0.88 |
| Friend support | 7.00 | -1.14 | 0.40 |  | 7.00 | -0.97 | -0.05 |  | 7.00 | -0.90 | -0.13 |  | 7.00 | -0.99 | 0.03 |
| Classmate support | 4.00 | -0.83 | 0.79 |  | 4.00 | -0.76 | 0.58 |  | 4.00 | -0.68 | 0.40 |  | 4.00 | -0.75 | 0.56 |
| Teacher support | 4.00 | -0.78 | 0.41 |  | 4.00 | -0.74 | 0.31 |  | 4.00 | -0.62 | 0.01 |  | 4.00 | -0.71 | 0.22 |
| Health complaints | 1.00 | 0.78 | -0.20 |  | 1.00 | 0.65 | -0.41 |  | 1.00 | 0.36 | -0.90 |  | 1.00 | 0.58 | -0.60 |
| Life satisfaction | 8.00 | -1.04 | 1.26 |  | 8.00 | -1.06 | 1.39 |  | 8.00 | -0.91 | 0.85 |  | 8.00 | -1.00 | 1.13 |
| *Notes.* SD: standard deviation; Skew.: skewness; Kurt.: kurtosis. Family and friend support involved a 1-7 range; classmate and teacher support, a 1-5 range; health complaints, a 1-5 range; life satisfaction, a 0-10 range. | | | | | | | | | | | | | | | |

| **Supplementary Material 2.** Trends in life satisfaction, stratified by country and region (*Health Behaviour of School-aged Children*, 44 countries and regions, 2013/14–2021/22). | | | | | | | | | | |
| --- | --- | --- | --- | --- | --- | --- | --- | --- | --- | --- |
|  | **2013/14** | |  | **2017/18** | |  | **2021/22** | |  | **ω² \| *d*** |
|  | *M* | *SD* |  | *M* | *SD* |  | *M* | *SD* |  |  |
| Albania | 8.22^a^ | 1.99 |  | 8.14^a^ | 2.03 |  | 8.66^b^ | 1.74 |  | **.014** |
| Armenia | 8.59^a^ | 1.87 |  | 8.34^b^ | 1.79 |  | 8.53^a^ | 1.68 |  | .004 |
| Austria | 7.95^a^ | 1.81 |  | 7.71^b^ | 2.04 |  | 7.60^c^ | 1.88 |  | .005 |
| Bulgaria | 7.85^a^ | 1.93 |  | 7.83^a^ | 1.99 |  | 7.33^b^ | 2.22 |  | **.011** |
| Canada | 7.37^a^ | 1.96 |  | 7.26^b^ | 1.99 |  | 7.06^c^ | 1.99 |  | .004 |
| Croatia | 7.91^a^ | 1.94 |  | 8.09^b^ | 1.85 |  | 7.98^a^ | 1.89 |  | .001 |
| Czechia | 7.20^a^ | 2.01 |  | 7.80^b^ | 1.74 |  | 7.61^c^ | 1.92 |  | **.012** |
| Denmark | 7.63^a^ | 1.72 |  | 7.68^a^ | 1.73 |  | 7.41^b^ | 1.81 |  | .005 |
| England | 7.39^a^ | 1.88 |  | 7.45^a^ | 1.86 |  | 6.94^b^ | 1.97 |  | **.013** |
| Estonia | 7.79^a^ | 1.85 |  | 7.73^a^ | 1.86 |  | 7.37^b^ | 2.05 |  | .009 |
| Finland | 7.69^a^ | 1.68 |  | 7.75^a^ | 1.77 |  | 7.70^a^ | 1.73 |  | .000 |
| Flanders (Belgium) | 7.07^a^ | 2.12 |  | 7.80^b^ | 1.42 |  | 7.72^c^ | 1.63 |  | **.027** |
| France | 7.33^a^ | 1.99 |  | 7.66^b^ | 1.79 |  | 7.19^c^ | 1.87 |  | **.012** |
| Germany | 7.38^a^ | 1.98 |  | 7.69^b^ | 1.74 |  | 7.60^c^ | 1.82 |  | .005 |
| Greece | 7.72^a^ | 1.78 |  | 7.54^b^ | 1.93 |  | 7.38^c^ | 2.05 |  | .005 |
| Greenland | 7.79^a^ | 2.17 |  | 7.87^a^ | 2.31 |  | 6.80^b^ | 1.98 |  | **.048** |
| Hungary | 7.54^a^ | 1.88 |  | 7.58^a^ | 1.93 |  | 7.38^b^ | 2.07 |  | .002 |
| Iceland | 7.73^a^ | 1.88 |  | 7.62^b^ | 1.97 |  | 7.43^c^ | 1.94 |  | .005 |
| Ireland | 7.59^a^ | 1.88 |  | 7.55^a^ | 1.89 |  | 6.98^b^ | 2.03 |  | **.019** |
| Israel | 7.98^a^ | 1.95 |  | 7.79^b^ | 2.41 |  | 7.58^c^ | 2.23 |  | .004 |
| Italy | 7.36^a^ | 1.95 |  | 7.58^b^ | 1.81 |  | 7.08^c^ | 1.94 |  | **.012** |
| Kazakhstan | – | – |  | 8.57^a^ | 1.84 |  | 8.25^b^ | 2.11 |  | *0.159* |
| Latvia | 7.35^a^ | 1.78 |  | 7.40^a^ | 1.93 |  | 7.12^b^ | 2.04 |  | .004 |
| Lithuania | 7.85^a^ | 1.92 |  | 7.91^a^ | 1.91 |  | 7.27^b^ | 2.02 |  | **.021** |
| Luxembourg | 7.44^a^ | 2.04 |  | 7.64^b^ | 1.85 |  | 7.54^ab^ | 1.84 |  | .002 |
| Macedonia | 7.66^a^ | 2.55 |  | 8.43^b^ | 2.08 |  | 7.84^c^ | 2.35 |  | **.020** |
| Malta | 7.61^a^ | 1.92 |  | 7.33^b^ | 2.08 |  | 7.18^c^ | 2.03 |  | .007 |
| Moldova | 8.25^a^ | 1.80 |  | 8.24^a^ | 1.70 |  | 8.07^b^ | 1.64 |  | .002 |
| Netherlands | 7.75^a^ | 1.64 |  | 7.77^a^ | 1.56 |  | 7.39^b^ | 1.77 |  | **.011** |
| Norway | 7.89^a^ | 1.80 |  | 7.90^a^ | 1.82 |  | 7.37^b^ | 1.84 |  | **.018** |
| Poland | 7.38^a^ | 2.13 |  | 7.48^a^ | 1.95 |  | 6.48^b^ | 2.41 |  | **.043** |
| Portugal | 7.49^a^ | 1.93 |  | 7.73^b^ | 1.79 |  | 7.57^a^ | 1.81 |  | .003 |
| Romania | 8.12^a^ | 1.91 |  | 8.33^b^ | 1.67 |  | 8.13^a^ | 1.69 |  | .002 |
| Russia | 7.30^a^ | 2.03 |  | 7.41^b^ | 2.04 |  | – | – |  | *0.054* |
| Scotland | 7.75^a^ | 1.88 |  | 7.62^b^ | 1.90 |  | 7.06^c^ | 1.97 |  | **.022** |
| Serbia | – | – |  | 8.25^a^ | 1.84 |  | 8.30^a^ | 1.84 |  | *0.027* |
| Slovakia | 7.40^a^ | 2.02 |  | 7.64^b^ | 1.85 |  | 6.40^c^ | 1.85 |  | **.072** |
| Slovenia | 7.73^a^ | 1.85 |  | 7.97^b^ | 1.77 |  | 7.44^c^ | 1.90 |  | **.014** |
| Spain | 7.85^a^ | 1.99 |  | 8.09^b^ | 1.74 |  | 7.30^c^ | 2.05 |  | **.019** |
| Sweden | 7.36^a^ | 1.91 |  | 7.46^b^ | 1.93 |  | 7.20^c^ | 2.00 |  | .002 |
| Switzerland | 7.73^a^ | 1.76 |  | 7.69^a^ | 1.85 |  | 7.39^b^ | 1.96 |  | .007 |
| Ukraine | 7.65^a^ | 1.89 |  | 7.68^a^ | 1.92 |  | – | – |  | *0.016* |
| Wales | 7.37^a^ | 1.90 |  | 7.62^b^ | 1.93 |  | 7.16^c^ | 2.05 |  | **.010** |
| Wallonia (Belgium) | 7.53^a^ | 1.97 |  | 7.56^a^ | 1.81 |  | 7.29^b^ | 1.94 |  | .004 |
| Sample | 7.64^a^ | 1.95 |  | 7.75^b^ | 1.91 |  | 7.44^c^ | 2.00 |  | .004 |
| *Notes*. When differing horizontally, superscript letters signal a statistically significant difference identified based on Tukey post-hoc tests (when data pertaining to all three waves were available) or on *t* tests (when data pertaining to two waves only were available). Omega squared (ω²) ≥ .010 are bolded; Cohen’s *d*s are italicized. | | | | | | | | | | |

| **Supplementary Material 3.** Mean scores of perceived social support pertaining to the countries that partook in two waves of data collection (*Health Behaviour of School-aged Children*, 44 countries and regions, 2013/14–2021/22). | | | | | | | | | | | | | | | | | | | |
| --- | --- | --- | --- | --- | --- | --- | --- | --- | --- | --- | --- | --- | --- | --- | --- | --- | --- | --- | --- |
|  | **Family support** [1-7] | | | |  | **Friend support** [1-7] | | | |  | **Classmate support** [1-5] | | | |  | **Teacher support** [1-5] | | | |
|  | 2014 | 2018 | 2022 | *d* |  | 2014 | 2018 | 2022 | *d* |  | 2014 | 2018 | 2022 | *d* |  | 2014 | 2018 | 2022 | *d* |
| Armenia |  |  |  |  |  | – | 5.52^a^ | 5.51^a^ | 0.00 |  |  |  |  |  |  |  |  |  |  |
| Canada | – | 4.98^a^ | 4.92^b^ | 0.03 |  | – | 4.81^a^ | 4.70^b^ | 0.07 |  |  |  |  |  |  |  |  |  |  |
| Denmark |  |  |  |  |  | – | 5.99^a^ | 5.70^b^ | **0.21** |  |  |  |  |  |  |  |  |  |  |
| Kazakhstan | – | 6.02^a^ | 5.82^b^ | 0.11 |  | – | 5.28^a^ | 5.39^b^ | 0.05 |  | – | 3.98^a^ | 3.90^b^ | 0.08 |  | – | 4.12^a^ | 3.91^b^ | **0.23** |
| Latvia |  |  |  |  |  |  |  |  |  |  | 3.69^a^ | – | 3.54^b^ | 0.17 |  | 3.79^a^ | – | 3.61^b^ | 0.19 |
| Lithuania | – | 5.79^a^ | 5.32^b^ | **0.28** |  |  |  |  |  |  |  |  |  |  |  |  |  |  |  |
| Macedonia |  |  |  |  |  | 5.54^a^ | 5.77^b^ | – | 0.14 |  |  |  |  |  |  |  |  |  |  |
| Poland |  |  |  |  |  |  |  |  |  |  | 3.82^a^ | – | 3.51^b^ | **0.37** |  | 3.65^a^ | – | 3.28^b^ | **0.40** |
| Russia | 5.31^a^ | 5.53^b^ | – | 0.12 |  | 4.84^a^ | 4.62^b^ | – | 0.11 |  | 3.64^a^ | 3.59^b^ | – | 0.06 |  | 3.60^a^ | 3.50^b^ | – | 0.10 |
| Serbia | – | 6.31^a^ | 6.01^b^ | **0.20** |  | – | 5.38^a^ | 5.53^b^ | 0.08 |  | – | 3.89^a^ | 3.95^b^ | 0.06 |  | – | 3.53^a^ | 3.53^a^ | 0.00 |
| Ukraine | 5.45^a^ | 5.65^b^ | – | 0.17 |  | 5.00^a^ | 4.86^b^ | – | 0.10 |  | 3.75^a^ | 3.50^b^ | – | **0.29** |  | 3.67^a^ | 3.54^b^ | – | 0.16 |
| *Notes*. When differing horizontally within a given source of perceived social support, superscript letters signal a statistically significant difference identified based on *t* tests. Cohen’s *d* ≥ .0.2 are bolded. | | | | | | | | | | | | | | | | | | | |

**Supplementary Material 4.** Temporal dynamics of perceived family support (mean scores [1-7] | *Health Behaviour of School-aged Children*, 44 countries and regions, 2013/14–2021/22).


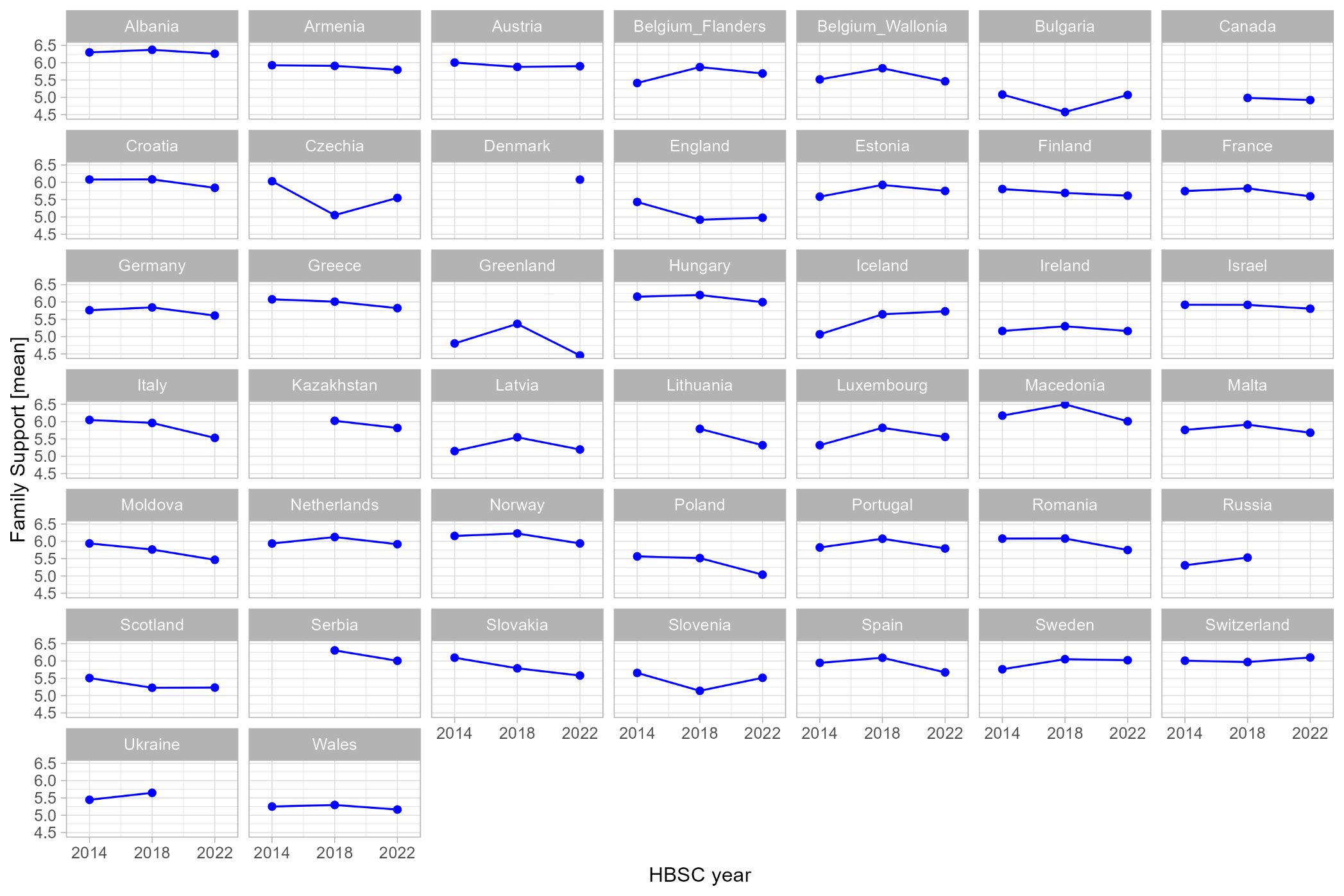


**Supplementary Material 5.** Temporal dynamics of perceived friend support (mean scores [1-7] | *Health Behaviour of School-aged Children*, 44 countries and regions, 2013/14–2021/22).


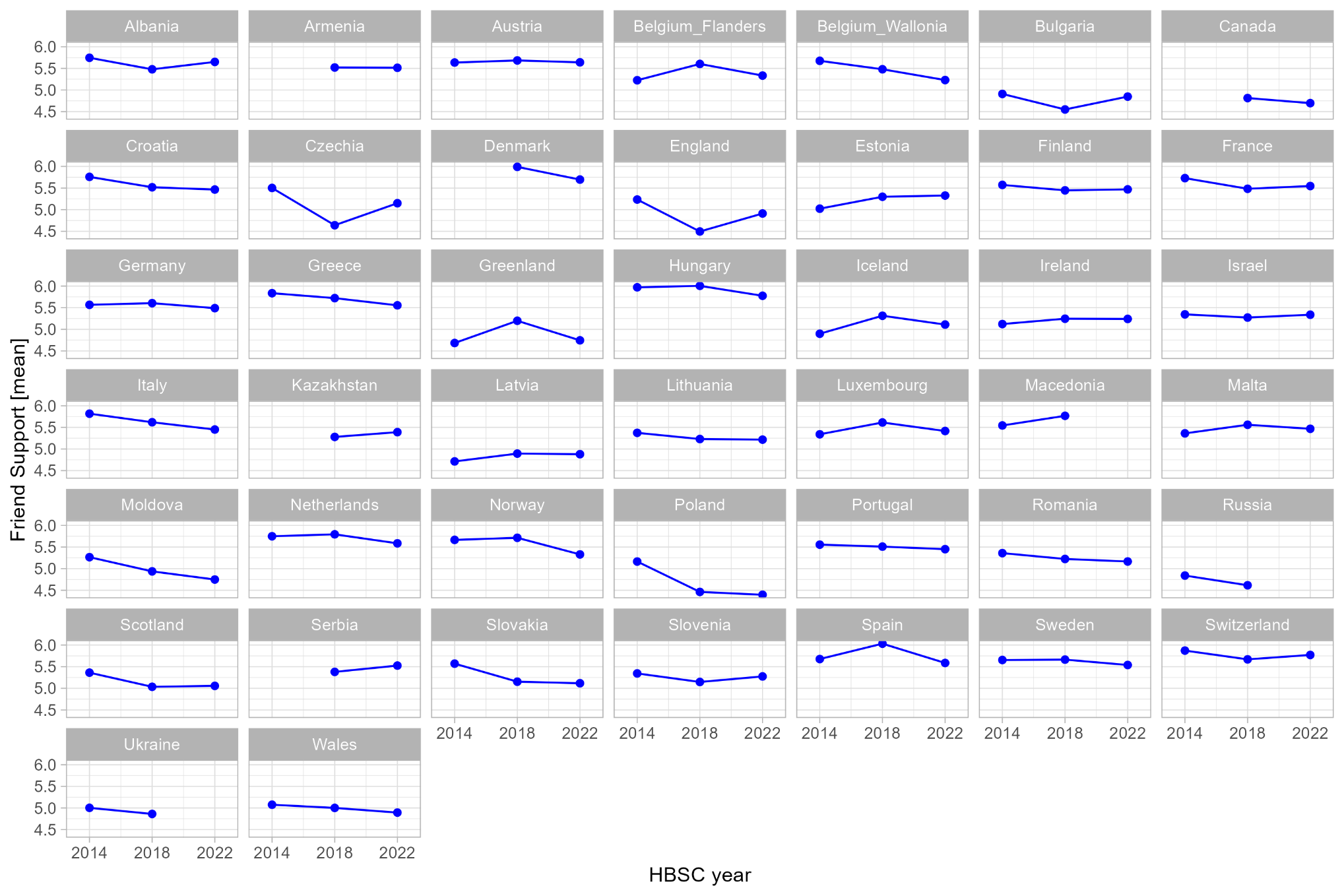


**Supplementary Material 6.** Temporal dynamics of perceived classmate support (mean scores [1-5] | *Health Behaviour of School-aged Children*, 44 countries and regions, 2013/14–2021/22).


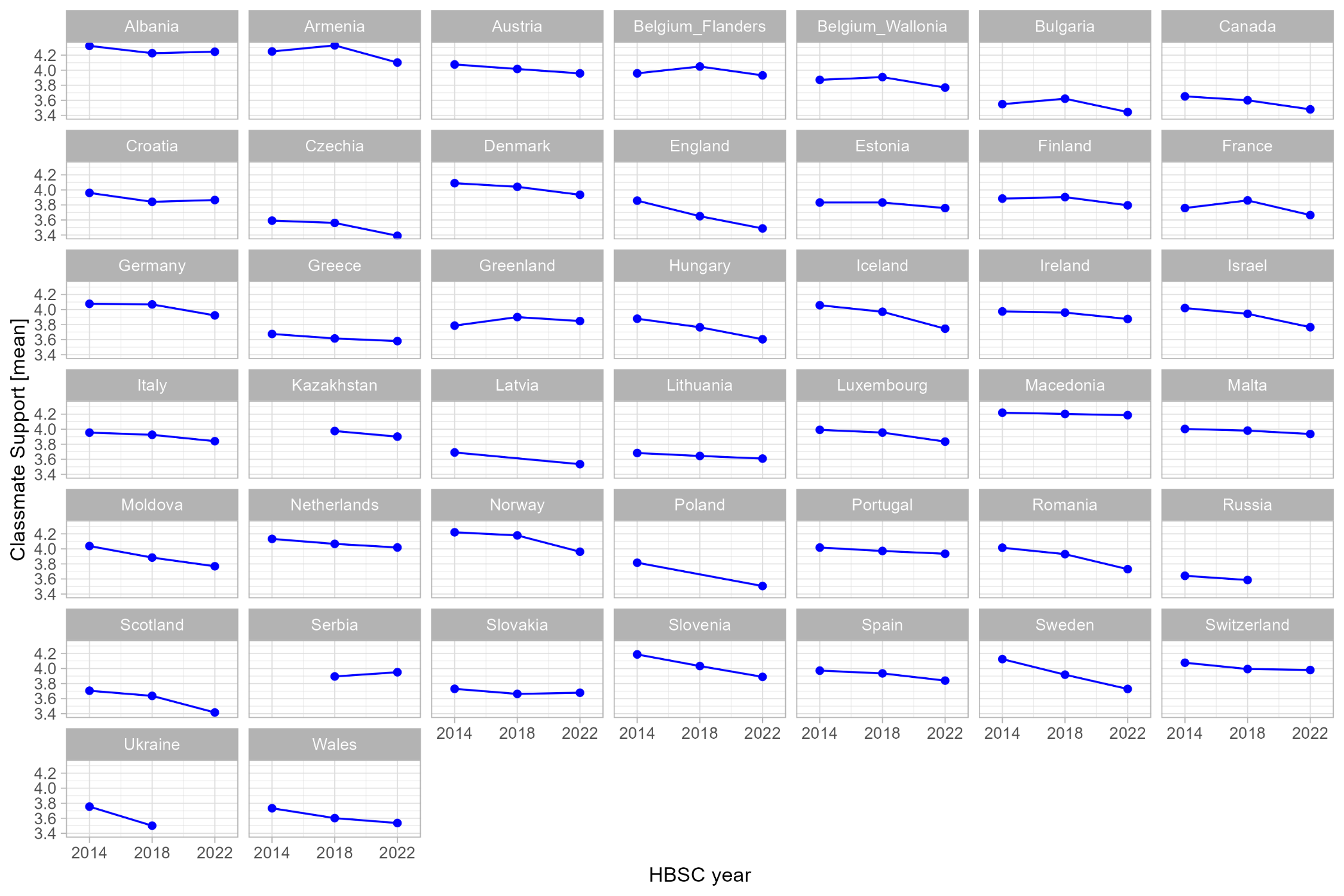


**Supplementary Material 7.** Temporal dynamics of perceived teacher support (mean scores [1-5] | *Health Behaviour of School-aged Children*, 44 countries and regions, 2013/14–2021/22).


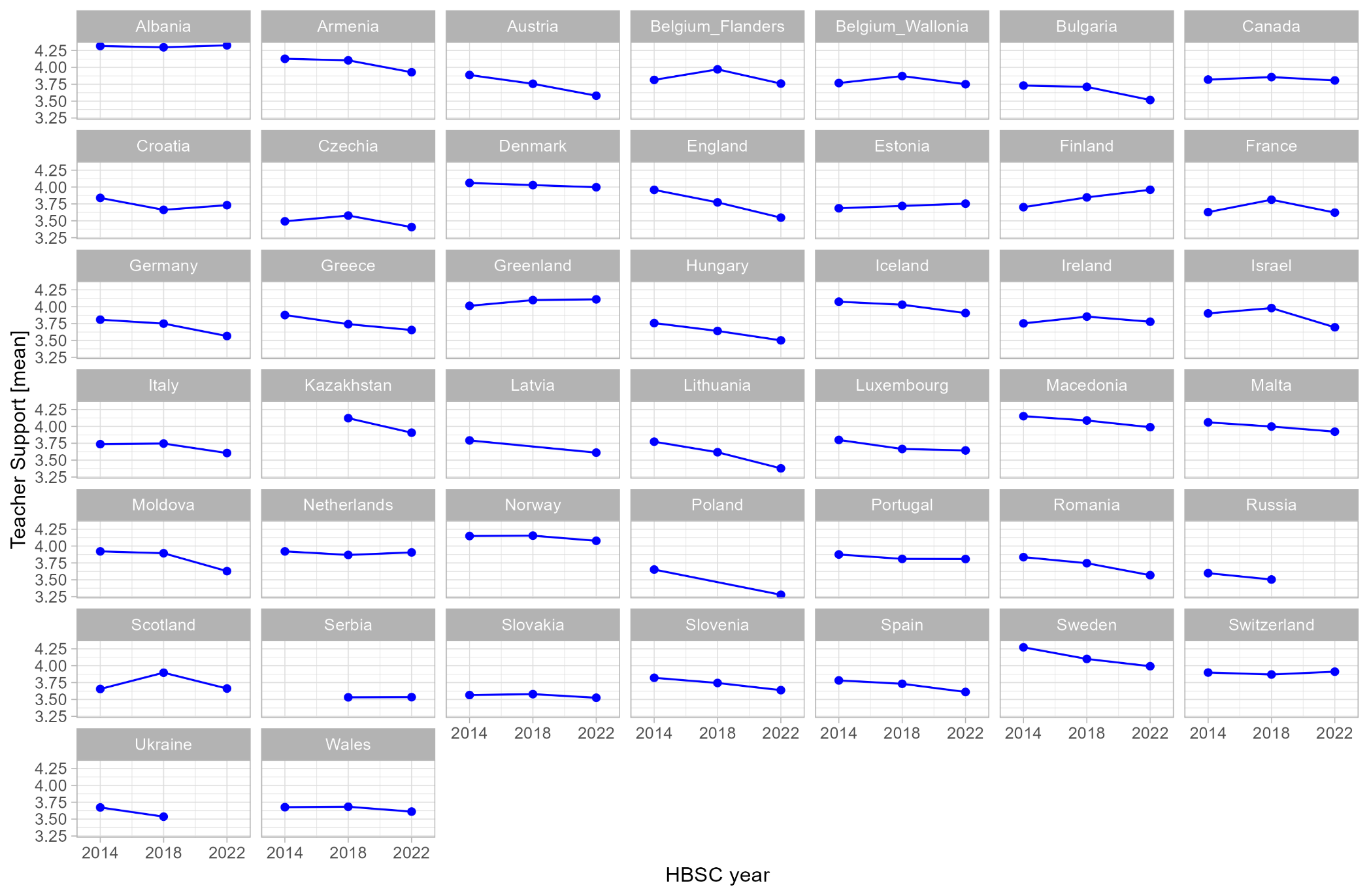


**Supplementary Material 8.** Temporal dynamics of life satisfaction (mean scores [0-10] | *Health Behaviour of School-aged Children*, 44 countries and regions, 2013/14–2021/22).


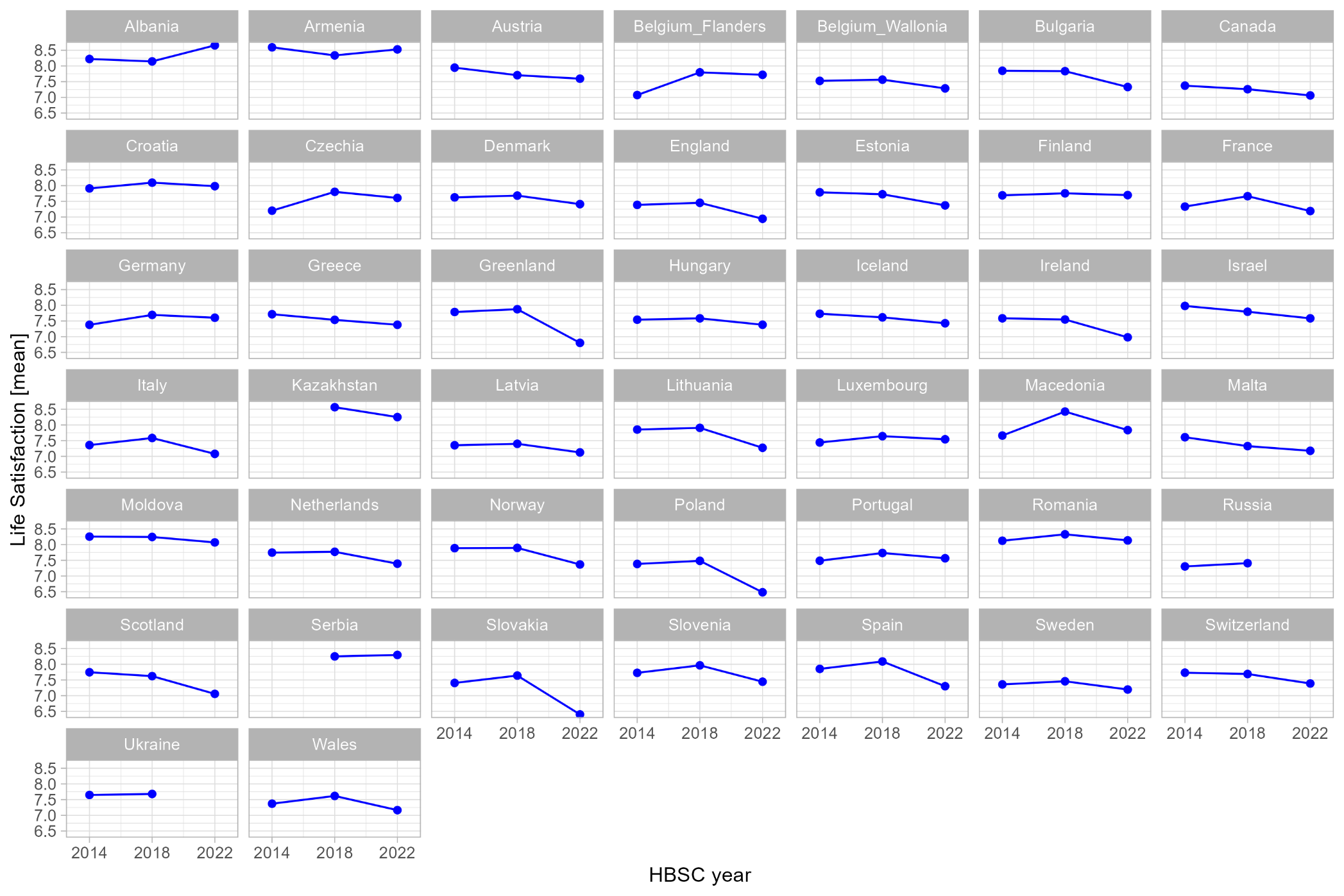

Supplement: Supplementary file 1 [file DataSheet1.docx]
